# A 338-year tree-ring oxygen isotope record from Thai teak captures the variations in the Asian summer monsoon system

**DOI:** 10.1038/s41598-020-66001-0

**Published:** 2020-06-02

**Authors:** Nathsuda Pumijumnong, Achim Bräuning, Masaki Sano, Takeshi Nakatsuka, Chotika Muangsong, Supaporn Buajan

**Affiliations:** 1 Faculty of Environment and Resource Studies, Mahidol University, Mahidol, Thailand; 2 Institute of Geography, Friedrich-Alexander University Erlangen-Nürnberg, Erlangen-Nürnberg, Germany; 3 Faculty of Human Sciences, Waseda University, Tokorozawa, Japan; 4 Nagoya University, Nagoya, Japan; 5 Innovation for Social and Environmental Management, Mahidol University, Amnatcharoen Campus, Amnatcharoen, Thailand

**Keywords:** Biogeochemistry, Palaeoclimate

## Abstract

A 338-year oxygen isotope record from teak tree-ring cellulose collected from Mae Hong Son province in northwestern Thailand was presented. The tree-ring series preserves the isotopic signal of the regional wet season rainfall and relative humidity. Tree-ring δ^18^O correlates strongly with regional rainfall from May to October, showing coherent variations over large areas in Southeast Asia. We reconstructed the summer monsoon season (May to October) rainfall based on a linear regression model that explained 35.2% of the actual rainfall variance. Additionally, we found that in the 19^th^ century, there was a remarkable drought during many years that corresponded to regional historic drought events. The signals of the June to September Indian summer monsoon (ISM) for the period between 1948 and 2009 were clearly found. Spatial correlations and spectral analyses revealed a strong impact of the El Niño-Southern Oscillation (ENSO) on tree-ring δ^18^O. However, ENSO influenced the tree-ring δ^18^O more strongly in the 1870–1906, 1907–1943, and 1944–1980 periods than in the 1981–2015 period, which corresponded to periods of weaker and stronger ISM intensity.

## Introduction

The Asian summer monsoon (ASM) is an important component of global monsoons and is one of the dominant summer rainfall regimes in the world. Changes in ASM activity have strong implications for the economy and livelihood of more than two billion people, which are directly or indirectly affected by the timing and amount of precipitation during the ASM season^1^. Therefore, understanding climate change and climate variability effects on the ASM is highly relevant. However, a clearer understanding of long-term monsoon variability is hampered by the scarcity of long instrumental climate records for the tropics. Thus, climate proxy records are needed to extend the existing instrumental climate series into the past to learn more about the long-term natural variability in the ASM and to analyze the recent climate change trends.

Thailand, a country located in tropical Southeast Asia, is governed by the ASM climate. The economy and environment of the region strongly depend on climatic conditions. Asian and Southeast Asian countries are home to a large population and are important global food producers^2^. As such, climate change could affect the intensity and frequency of rainfall patterns, which could seriously impact food production.

Among other climate proxies, tree rings have the advantages of an annual resolution and precise dating control^3^. One restriction pertaining to dendrochronological studies within the Southeast Asian region is the scarcity of tree species that produce clear annual growth rings. Although a number of tree species in South Asia have recently been found to produce annual tree rings and can produce tree-ring chronologies that are useful for climate reconstruction^4^, mostly teak (*Tectona grandis*, L.f), they have so far been successfully used to produce long tree-ring chronologies used for climate reconstructions from the Asian tropics due to their wide distribution; teak was also been used as construction timber in historical buildings. However, previous studies^5–9^ provided climate reconstructions based on teak tree-ring records, which did not pass some verification tests. As an alternative method, stable isotopes in tree rings have been examined worldwide, especially in tropical regions^10–12^ to gain information about variations in the hydrological system^13^.

To this end, the oxygen isotope ratios (δ^18^O) in tree-ring cellulose are increasingly being used as a tool to obtain retrospective insights into ecophysiological processes^14^. One advantage of using the δ^18^O in tree-ring cellulose compared to tree ring width is that δ^18^O lacks internal trends related to juvenile effects^15^. In addition, tree-ring δ^18^O first reflects the isotopic composition of the source water, which is then altered by leaf enrichment^16^.

Hence, δ^18^O in tree-ring cellulose can provide insights into the causes behind the variations in moisture sources^17,18^. Existing tree-ring stable oxygen isotope studies in temperate climate zones are numerous^19–21^. The study of oxygen isotopes in tropical regions in South America is still more abundant than those in Southeast Asia, such as Costa Rica^11,12^, and in Bolivia^13^, the number of existing tree-ring studies using stable isotopes within Southeast Asia remains small^22–25^. This lack of studies is partly due to the lack of suitable, old specimens but also due to the lagged development in terms of tropical dendroecological discoveries of new tree species suitable for tree-ring studies. In this study, we produced the longest oxygen isotope chronology currently available from tree-ring cellulose of teak and examined the influence of local and regional climate parameters on oxygen isotope ratios (δ^18^O) to improve our understanding of ASM variability during the past 338 years.

## Results

### Teak tree-ring δ^18^O (δ^18^O_tr_) chronology

**Figure 1:**
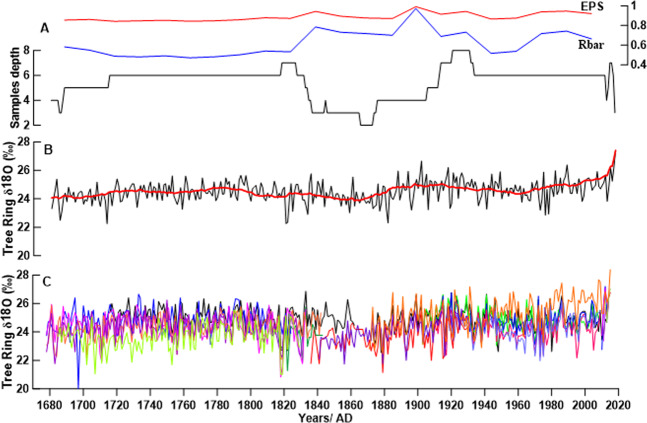
Sample depth, running EPS, and Rbar statistics of the mean isotope chronology (**A**) Thai teak tree-ring δ^18^O chronology (black line), 11-year low pass filter (red line) (**B**), and individual teak tree-ring δ^18^O series (**C**).

Results indicated significant intercorrelations for the most of the individual tree-ring δ^18^O (δ^18^O_tr_) series and permitted for calculating the mean regional site chronology. Figure 1 shows the annually resolved δ^18^O_tr_ mean values of tree-ring cellulose from 21 trees for the period AD 1678–2015 along with the running expressed population signal (EPS) and inter-series correlation (Rbar) statistics, through the use of 30-year windows, and when lagged 15 years (Fig. 1). The Rbar statistic for these data spans a range of 0.4–0.81, and EPS is in the range of 0.7–0.95. For our δ^18^O_tr_ series, the EPS value of ≥0.85 is generally accepted but fell below this criterion is associated with diminished the sample size.^26^. Buras^27^ explained that EPS indicates how good a finite sample of tree-ring data is for an infinite population. Therefore, values that are lower than 0.85 do not mean that the chronology is unreliable. Due to the tree-ring δ^18^O study, the number of samples taken is not as high as those for the tree ring width study. Therefore, at some times, an EPS value lowers than 0.85 does not mean that the value is unreliable. Teak δ^18^O_tr_ values ranged between 20.81 and 26.90‰, and the long-term average was 24.44‰.

### Teak δ^18^O_tr_ and local and regional climate data

**Figure 2:**
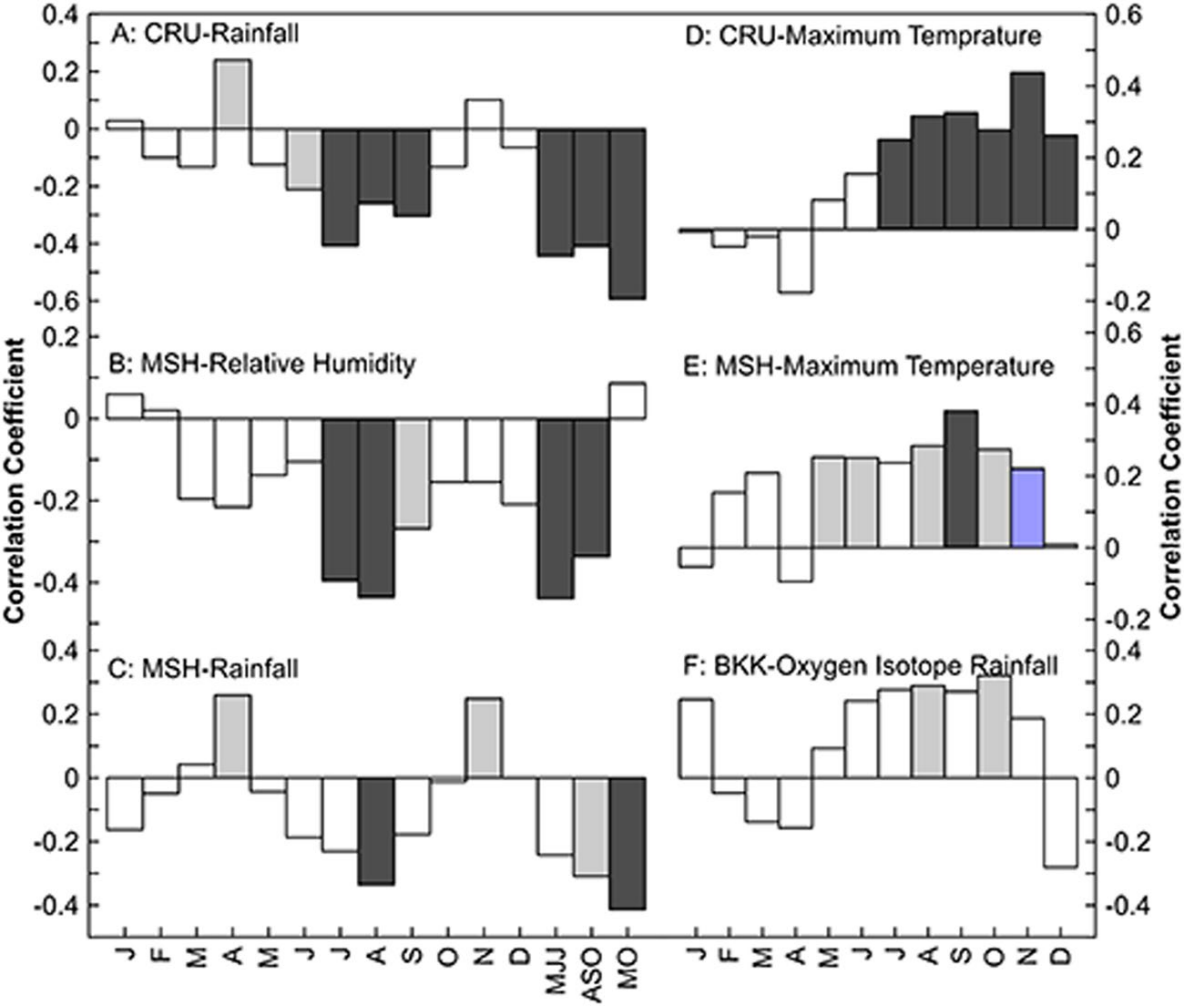
Correlations between tree-ring δ^18^O and rainfall obtained from the CRU TS4.03 during the period 1901–2015 (**A**), relative humidity (%) obtained from the Mae Hong Son meteorological station during the period 1950–2015 (**B**), rainfall obtained from the Mae Hong Son instrumental station during the period 1911–2015 (**C**), mean maximum temperature obtained from the CRU TS4.03 during the period of 1901–2015 (**D**), mean maximum temperature at Mae Hong Son meteorologica station during the period of 1951–2015 (**E**), and δ^18^O in rainfall in Bangkok (**F**). Black bars indicate correlations that are significant at the 99% level of confidence; light blue bars indicate correlations significant at the 95% level of confidence.

Figure 2 shows the relationship among δ^18^O_tr_ and rainfall, humidity, and temperature at the Mae Hong Son meteorological station (hereinafter named the local climate), δ^18^O_rain_ at Bangkok Station and CRU T4.03 rainfall and temperature (hereinafter named the regional climate). The δ^18^O_tr_ has a negative correlation with the local rainfall (May–October (M–O), *r* = *−*0*.413, p* < *0.001*), local humidity (May–June–July (MJJ), *r* = *−0.439, p* < *0.001*) and regional rainfall (M–O, *r* = *−0.593, p* < *0.001*).

**Figure 3:**
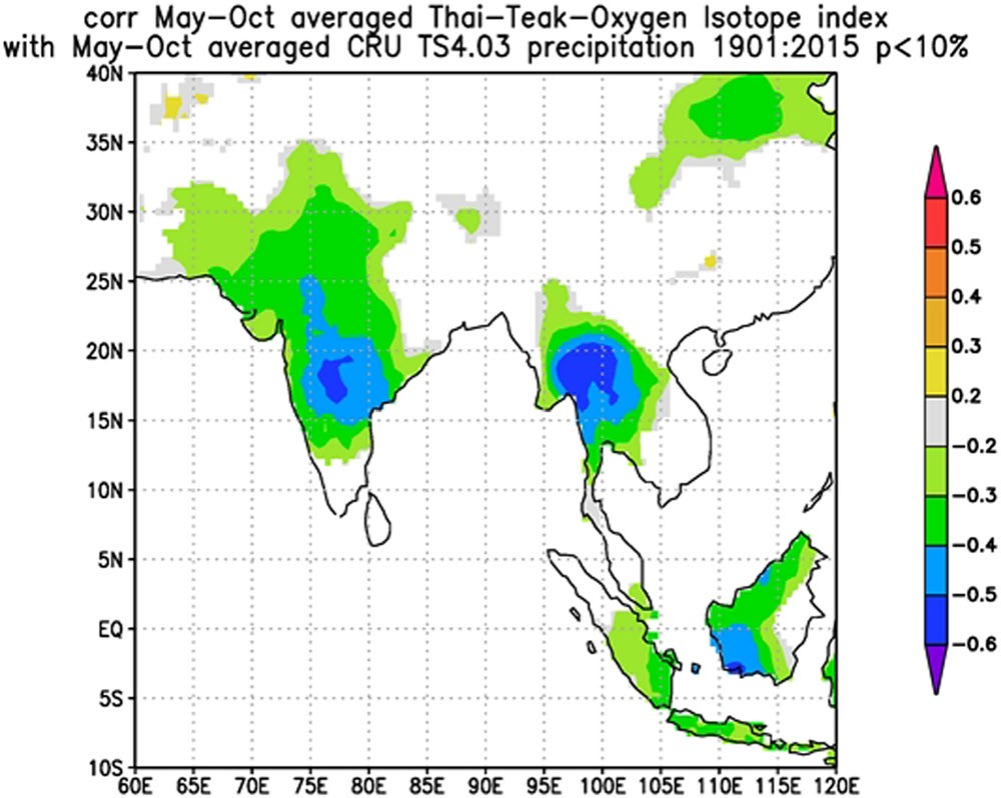
Spatial correlation pattern of Thai Teak δ^18^O_tr_ vs. CRU TS4.03 May–October precipitation.

A spatial correlation analysis was performed to demonstrate the relationship between δ^18^O_tr_ and the amount of precipitation in the tropics. Regions showing significant negative correlations with the δ^18^O_tr_ series appeared over wide areas in Southeast Asia as well as in the eastern parts of the Indian subcontinent. The correlation was strongest over northwestern Thailand and decreased toward the east (Fig. 3).

### Teak δ^18^O_tr_ and monsoon indices

The significant negative relationship between δ^18^O_tr_ and ISM and the Webster and Yang Monsoon Index (WYM) was highest from June to August (JJA) (*r* = *−0.544, p* < *0.001*) and highest from June to September (JJAS) (*r* = *−0.459, p* < *0.001*) (data not shown).

### Spectral analyses

The spectral analysis revealed significant peaks (*p* < *0.01*) at years 100, 4 and 2 (Fig. S1) for cyclicity. Notably, short cycles of 4–7 years correspond to the typical ENSO frequency band. The significant periodicity band with a frequency of 100 years may be related to the Pacific Multidecadal Oscillation^28^ and long-term summer monsoon variability^29^.

### Global correlation between δ^18^O and sea surface temperature

We found significant positive relationships between our δ^18^O_tr_ and the multivariate ENSO index (MEI). Correlations with the extended multivariate ENSO index (MEI_ext range of 1871–2005) were similar to MEI, in which the relationship began in May, with September showing the highest positive correlation (*r* = 0.565, *p* < 0.001). The same relationship was found between δ^18^O_tr_ and both Niño3.4 and Niño4, with the highest positive correlation occurring in October for Niño4 (*r* = *0*.577, *p* < *0.001*) (Fig. S2). Correlations between δ^18^O_tr_ and Dipole Mode Index (DMI) over the entire period (1870–2015) revealed significant positive relationships from June to October, with October being the month that had the highest correlation (*r* = *0.453, p* < *0.001*). Furthermore, a positive relationship between δ^18^O_tr_ and Pacific Decadal Oscillation (PDO) is found from July to December, with the highest correlation in August (*r* = 0.258, *p* < 0.005). Our δ^18^O_tr_ and Palmer Drought Severity Index (PDSI) showed significant negative correlations over the whole year, with the highest correlations being from October to December (*r* = −0*.449*, *p* < *0.001*).

### Reconstruction of May to October (M–O) rainfall

**Figure 4:**
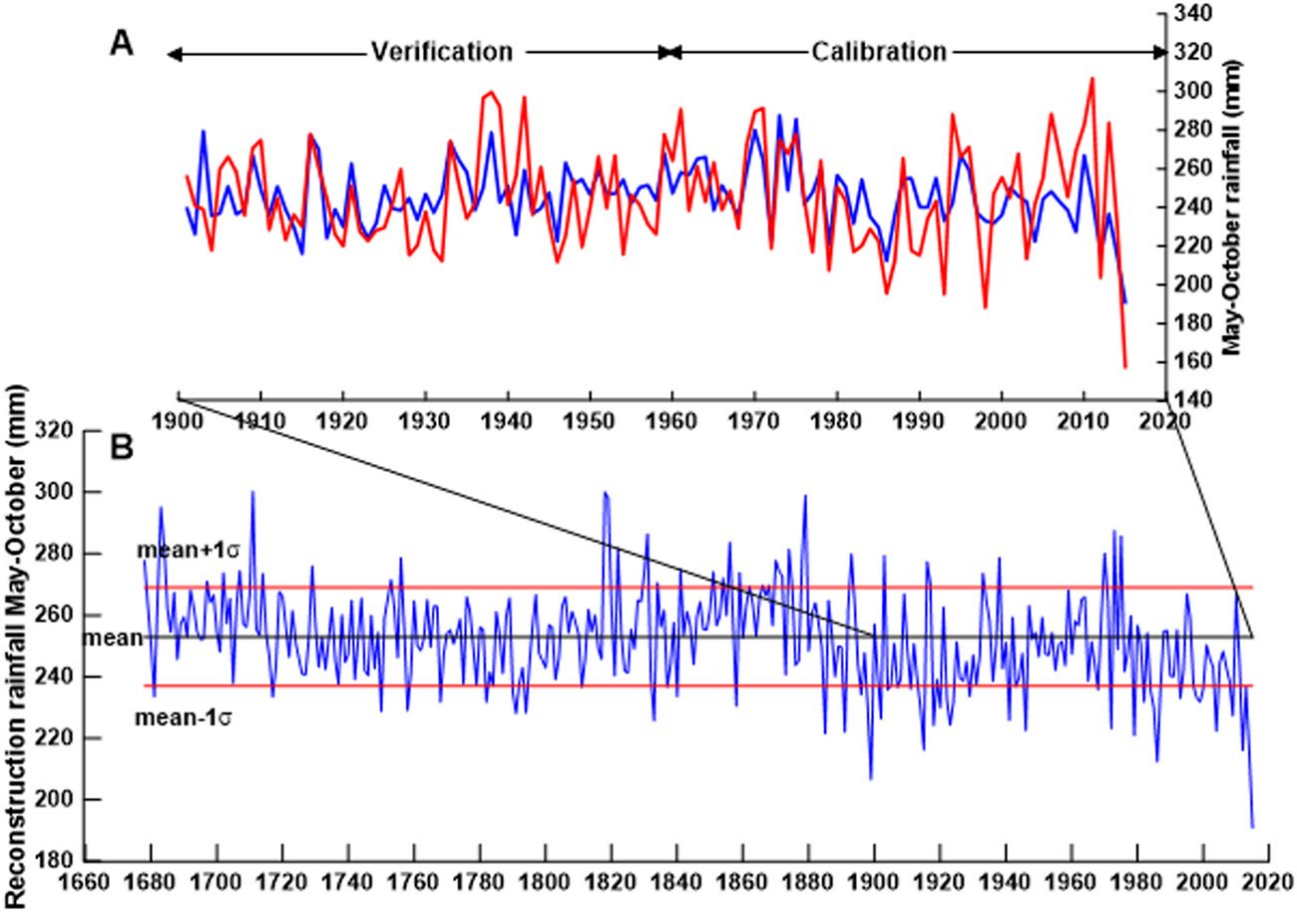
May to October rainfall (mm), red line is actual CRU TS4.03 rainfall (mm) and blue line is reconstruction May-October rainfall (mm) (**A**), reconstruction rainfall May–October black line is the average rainfall of 253 mm, read line (mean + 1σ) is 269 mm, and (mean − 1σ) is 237 mm (**B**).

The correlation between δ^18^O_tr_ and regional climate precipitation of M–O showed the highest value (*r* = −*0.593, p* < *0.001*); therefore, precipitation during the ASM season was targeted for climate reconstruction. We used a simple regression model to develop a transfer function, which is shown as follows: P_MO_ = 772.985 + (−21.250) * δ^18^O_tr_, where P_MO_ represents May–October rainfall. We divided this period into two subperiods, 1904–1959 and 1960–2015, for crosswise calibration and verification. The verification and calibration statistics are shown in Table 1. The values for Reduction of Error (RE) and Coefficient of Efficiency (CE) are positive in both subperiods, indicating the validity of the oxygen isotope record as a climate estimate^30^. Finally, we reconstructed the precipitation from May–October based on the full dataset for calibration. The linear regression model explained 35.20% of the actual variance in the May-October precipitation (Fig. 4).

**Table 1:** Calibration and verification of May to October precipitation reconstruction.

Calibration				Verification				RE	CE
Period	r	r^2^	ST	period	r	r^2^	ST		
1904–1959	0.514	0.264	36/−20	1960–2015	0.643	0.413	35/−21	0.386	0.385
1960–2015	0.643	0.413	37/−19	1904–1959	0.514	0.264	35/−21	0.229	0.227
1904–2015	0.593	0.352	72/−40						

ST = sign test, RE = reduction of error, CE = coefficient of efficiency

Significant level at p < 0.05 level

Figures and captions:

## Discussion and conclusions

### Teak oxygen isotope chronology characteristics

A 338-year-long teak oxygen isotope chronology for the period of AD 1678–2015 was developed, including data from 21 individual living teak trees from the Mae Hong Son Province, northwest Thailand. Instead of pooling the tree-ring material from several trees as mentioned above, we constructed the chronology by averaging the isotope series from individual trees that were well intercorrelated, allowing precise dating control on all individual δ^18^O_tr_ values. For this approach, our chronology is covered by at least 4 trees after 1680; however, there is one particular period (ca. 1860–1870) where the chronology shows low replication. It is possible that the period was one of the heavy teak concessions, so old trees did not remain (Fig. 1).

### Possible mechanisms for the strong precipitation signal in δ^18^O_tr_

Our δ^18^O_tr_ chronology exhibits a stable inverse relationship with the local and regional rainy season (May to October) precipitation (*r* = *−0.413, p* < *0.001*, n = 65 and *r* = *−0.593, p* < *0.001*, n = 112, respectively), probably as a result of the well-known “amount effect”^31^, and a positive relationship with the maximum temperature from June to December (highest in November, *r* = *437, p* < *0.001*, n = 112) during the dry season. It is possible that the source water signal of the soil is dominated by seasonally changing δ^18^O signatures in precipitation. As our study trees grew on mountain slopes, any groundwater impact on the tree-ring oxygen isotope signature can be excluded. In addition, teak has a shallow root system^32^ and grows well on well-drained soils. Thus, variations in oxygen isotope series in teak wood are expected to reflect the variations in oxygen isotope ratios in seasonal rainfall, which is affirmed by the significant positive correlation between δ^18^O_tr_ and annual δ^18^O in rainfall from the Global Network of Isotopes in Precipitation (GNIP) station in Bangkok (August *r* = *0.289*, *p* < *0.045*, n = 48, October r *r* = *0*.321, *p* < *0.030*, n = 46). Duy *et al*.^33^ concluded that the variation in oxygen isotopes in rainwater is 70% controlled by regional moisture regimes compared to local climatic conditions (30%), and regional and local factors vary in importance seasonally and have a large influence on the isotopic composition of rainfall. According to our results, local rainfall and regional rainfall are related to δ^18^O_tr_ in the same direction, and seasonal rainfall quite clearly controls the signature in δ^18^O_tr_. However, it is also clear that local precipitation throughout the rainy season (MO) has less relation to δ^18^O_tr_ than regional rainfall. Rainfall in the Mae Hong Son Province is clearly influenced by the Indian Ocean at the beginning of the rainy season (MJJ) and by the South China Sea at the end of the rainy season (ASO). The results of this study have been confirmed by the studies of Cai *et al*.^34^, Muangsong *et al*.^24^, and Wei *et al*.^35^. We showed the moisture trajectory in May, which is the source of moisture from the Indian Ocean, in September, which is the source of moisture from the South China Sea (Fig. S3) and the wind vector from 1981–2010 during May to July, August to September, and November to December. It is clear that the direction of the wind changed from the beginning of the rainy season to the dry season (Fig. S4). However, the regional climate covers more extensive areas, which causes fluctuations in δ^18^O_rain_ and the enrichment of oxygen isotopes in leaves until they accumulate in trees. Therefore, it is found that regional rainwater throughout the rainy season is associated with higher δ^18^O_tr_ than local rainwater. Nevertheless, more research on the relationship of δ^18^O_rain_ and amount of rainfall in the regions influenced by the Asian monsoon pointed out that it was controlled by convective heat transfer and terrain^36,37^. Therefore, we find that not every month of rainfall is associated with δ^18^O_tr_.

Furthermore, our results correspond to the studies of Brienen *et al*.^13^ and Volland *et al*.^38^, which used δ^18^O_tr_ in tree rings of *Cedrela spp*. from the Amazon basin and its surroundings and found significant positive correlations between δ^18^O_tr_ and δ^18^O in rainfall. The climate signals captured by our isotope record also agree with the studies of Muangsong *et al*.^24,25^ and Schollaen *et al*.^23^. During the transitional months from the end of the dry season to the beginning of the rainy season (April), teak cambium becomes active^28^. However, evaporation during this time of the year is high, leading to ^18^O-enriched leaf water. Hence, we found stronger correlations between δ^18^O_tr_ and air humidity (July, August, and September, *r* = *−0.396, p* < *0.001*, n = 66, *r* = *−0.436, p* < *0.001*, n = 66, *r* = −*0.269, p* < *0.029*, n = 65). Tree-ring δ^18^O values in this region are not only controlled by total (or annual) rainfall but also by monthly and seasonal rainfall isotopic signatures. Hence, dominant variations in monthly and/or seasonal inputs of rainwater isotope signals are related to different humidity sources, which can be assigned values of δ^18^O_tr_ in this region and may also result in different δ^18^O_tr_ values, which may be the same species^39^ or trees that grow in nearby areas^22,24^. In addition, an important process affecting δ^18^O_tr_ is the fractionation of the oxygen isotope that occurs in leaves through the evapotranspiration process compliance with the maximum temperature control fluctuations in oxygen isotopes during warmer conditions, which enhances the evaporation of the soil water and increases δ^18^O in the source water^40^, resulting in the abundance of the leaf water isotope^41^.

### Comparison between δ^18^O_tr_ and other proxies in nearby areas

The relationship between our teak δ^18^O_tr_ and teak δ^18^O_tr_ from the Phrae Province, which is approximately 400 km away, was *0.501, p* < *0.001*^42^, and Myanmar teak δ^18^O_tr_ was *0.566, p* < *0.001*^43^. We also found a relationship between our teak δ^18^O_tr_ and *Pinus merkusii* δ^18^O from Mae Hong Son (*0.646, p* < *0.001*)^44^ and from Umpang, Tak Province (*0.522, p* < *0.001*)^45^. This correlation coefficient was higher than the correlation found with the *Pinus kesiya* oxygen isotope series studied by Zhu *et al*.^46^, indicating that there was a common moisture source for the studied teak and *Pinus merkusii* trees. This has important implications for further studies on stable oxygen isotopes for both species to extend the existing records. It is interesting because the *Fokienia hodginsii* tree-ring δ^18^O from Vietnam (*r* = *0.328, p* < *0.001*)^47^ and the same species from Laos (*r* = *0.174, p* < *0.005*)^48^ correlated with our teak δ^18^O_tr_. The oxygen isotopes in tree rings are a great tool to study the hydrology cycle in Southeast Asia (Fig. S5).

In addition to the tree-ring δ^18^O that are extensively used in the study of monsoon dynamics, stalagmites in Thailand have also been studied for monsoon dynamics. We found that the teak δ^18^O_tr_ has a positive relationship with high-resolution oxygen isotope record of stalagmites from Klang Cave in southern Thailand^49^ after running a 31-point smoothing filter (*r* = *0.328, p* < *0.001*) as well as growth rate profile, derived from stalagmite NJ-0901, from Nam Jang Cave in Mae Hong Son province of northwestern Thailand^50^ based upon data smoothed with a 7-point running-average filter (*r* = *0.430*, *p* < *0.001*) (Fig. S6). Therefore, it is likely that we will gather proxies to be used to study the dynamics of the monsoon to unravel the complexity of the monsoon (Fig. S6).

### Reconstruction of summer precipitation during the past 338 years

Our May to October precipitation reconstruction revealed that over the entire period from 1678–2015, summer precipitation in northwest Thailand had decreased, and the reconstructed long-term average of May–October (rainy season) average precipitation was 253 mm. The dry period mean minus the standard deviation is equal to 237 mm, and the wet period mean plus the standard deviation is equal to 269 mm. We found that during the 17^th^ and 18^th^ centuries, drought occurred during 8 years, respectively. In the 19^th^ century, there was drought during 19 years. In the 17^th^, 18^th^, and 19^th^ centuries, wet years occurred during 6, 10 and 1 years, respectively. Our May–October rainfall reconstruction corresponds to the findings of Xu *et al*.^44,45^ using pine tree-ring oxygen isotopes, from both the Mae Hong Son and Tak Provinces. It was found that during the 17^th^ century, rainfall tended to decrease, and the drought period continued more often than during periods of heavy rainfall. The mechanism that may support drought in Southeast Asia is the southward shift in the Intertropical Convergence Zone (ITCZ). The results of the rainfall reconstruction using stalagmite δ^18^O from Klang Cave^49^ found that the ITCZ has moved southward since the 18^th^ century, and the most noticeable period is the 1980s.

In addition, our results are in line with the findings of Buckley *et al*.^6^, who built a teak growth index of living trees and stumps that covered 448 years. In particular, Buckley *et al*.^6^ proposed a relationship between teak growth and PDSI and highlighted the drought that was recorded by the teak index in the early and mid-1700s. Our study also found a significantly negative correlation between the δ^18^O_tr_ of teak and PDSI, showing higher correlation coefficients than the teak ring width index. In addition, these findings are consistent with other reconstructed mega drought events, especially those found in the 18^th^ century, such as the Strange Parallels drought (1756–1768) and the East India drought (1790–1796)^51^. Anderson *et al*.^52^ found that during almost the entire 18^th^ century, the weakening of the southwest monsoon was a result of cooler North Atlantic sea surface temperatures (SST).

### Large-scale drivers of interannual to decadal variations in tree-ring oxygen isotopes

The significant positive relationships of our δ^18^O_tr_ with MEI (1950–2015), MEI extended (1871–2005), Niño3.4 (1870–2015) and Niño4 (1870–2005) point to a strong impact of ENSO on rainfall variability in northwest Thailand.

We calculated the relationships between δ^18^O_tr_ and Niño4 SSTs over different periods. During the four periods of 1870–1906, 1907–1943, 1944–1980, and 1981–2015, δ^18^O_tr_ and SSTs showed strong positive correlations. The most affected time was in the period of 1870–1906, the relationship between δ^18^O_tr_ and Niño4 shows a decrease in the positive relationship, and the month that appears to be somewhat delayed but is still significant is between 1981–2015. In general, the IM was strongly influenced by the ENSO during the first half of the 20^th^ century, whereas since the 1980s, the IM has been increasingly influenced by the Indian Ocean Dipole (IOD)^53^. Pratarastapornkul^54^ proposed that the IOD phenomenon affects annual rainfall in Thailand and that its impact varies depending on the combined effect and strength of the ENSO and IOD in the Pacific Ocean. As such, it is likely that excess rainfall and severe floods are to be expected in most regions of Thailand in cases of strong IOD events that combine with weak El Niño events.

The El Niño-Southern Oscillation (ENSO) is a major driver of global climate variability. The ENSO also interacts with other modes of climate variability, such as the Indian summer monsoon rainfall (ISMR). The easterly trade winds and SST gradients across the equatorial Pacific undergo a regime change, with enhanced trade winds and significant cooling (warming) over the tropical eastern (western) Pacific in the later period. Previous research has shown that the relationship between the ISRM and SSTs is variable^55,56^. The strongest relationships were found on short timescales (interdecadal periodicity, 2–7 years) or decadal periodicity (10.5 years) but with varying significance levels^57^. Several studies have examined the relationships between the ISM and ENSO phenomenon and/or variations in SST and sea surface pressure (SSP) in the Pacific Ocean^58^. This result suggests that δ^18^O_tr_ records multiple ENSO phenomena. Several studies^59,60^ have pointed to the fact that the influence of the ENSO phenomenon on the ASM varies over time.

Similar influences of ENSO on the isotopic signature in tree rings were observed in North Laos^61^, Thailand, Indonesia^62^, and China^63,64^. However, the strength of the ENSO influence was variable throughout the study period. In Bolivia, a reduced influence of ENSO during 1950–1974 coincided with periods of lower variance in the Southern Oscillation Index. However, it was also found that the lowest correlations with Niño 4 events occurred between 1981 and 2015. There are multiple factors that make the relationship between our δ^18^O_tr_ and El Niño lower. One probable factor is caused by rapid traversing of the Intertropical convergent zone (ITCZ) and/or the teleconnections with the northern Atlantic thermohaline circulation, which could weaken the Asian monsoon through the air-sea connection^65^. Singhrattna *et al*.^66^ found that in the past decade, Pacific SSTs have had a negative relationship with the summer monsoon in Thailand, but this relationship weakened prior to 1980 due to changes in the Walker circulation over the Thailand-Indonesian region. Therefore, variations in δ^18^O_tr_ with El Niño periods could come from several factors and require a more in-depth analysis.

Ashok *et al*.^67^ explained the impact of the IOD on the IM and ENSO during the period of 1958–1997, stating that the IOD and ENSO have an integral effect on the Indian summer rainfall (ISR). Whenever the ENSO-ISR correlation was low (high), the IOD-ISR relationship was high (low). However, Ashok *et al*.^68,69^ found that the IOD is a physical mode of the tropical Indian Ocean and that the evolution of the IOD is mostly independent from the Pacific’s influence^70,71^.

Based on the results presented in Fig. S2(A–C), SSTs in the central Pacific Ocean and Indian Ocean during the periods of 1870–2015, 1870–1942 and 1943–2015 showed that SSTs of both oceans are highly correlated with our δ^18^O_tr_. Positive IOD phases occurred in 1961, 1963, 1972, 1982, 1983, 1994, 1997, 2006, and 2012. Furthermore, we found that Thailand’s rainy season precipitation was below average in 1972, 1979, 1982, 1984, 1985, 1986, 1993, 1998, 1999, 2004, 2009, 2012, 2014, and 2015. Bridhikitti^72^ investigated the connection of ENSO/IOD with Thai rainfall anomalies during the period of 1980–2011 based on instrumental data from 17 locations. He found that the effect of ENSO on summer monsoon rainfall was not obvious, but the negative (positive) IODs in October, November, and December corresponded with La Niña (El Niño) signals, which may be seen in increased (decreased) rainfall on the southeast coast during the months of December, January and February.

Severe IOD events in the summer monsoon season could affect rainfall in northern Thailand in the following year. Chansaengkrachang *et al*.^73^ studied the time lags between IOD and rainfall over Thailand during the period of 1979–2008 based on rainfall data derived from 80 meteorological stations spread throughout Thailand. It was found that signal consistency in years of strong IOD showed an offset of approximately 11 months. This indicates that the IOD leads the rainfall by approximately two months. In addition, Hochreuther *et al*.^74^ found positive correlations between Sikkim larch (*Larix griffithii*) δ^18^O_tr_ during strong positive IOD phases in southeast Tibet, when heavy rains had occurred in the western part of the Indian Ocean and less rain/drought occurred in Indonesia and Australia.

Clearly, the ISM variability is complex and related to many phenomena, such as ENSO, differences in surface temperatures in the Indian Ocean (IOD) and the difference in North Atlantic SSTs^75^. Our δ^18^O_tr_ captured the season of the ISM. Our δ^18^O_tr_ showed a significant positive correlation with the ENSO phenomenon. As such, the creation of a network of oxygen isotope tree-ring cellulose chronologies to cover a wider area and extend the length of δ^18^O_tr_ is needed and would help us to better understand long-term ASM variability and its interrelationships with other atmospheric circulation patterns and climate forcing factors. This holds particularly true for Thailand, since the currently existing tree-ring δ^18^O are all from the Mae Hong Son Province in Northwest Thailand. To better understand the influence of the monsoon nationwide, analyzing specimens from other areas and examining the consistency of monsoon influences over the entire country is needed. Additionally, there are good possibilities to further extend the length of the existing tree-ring δ^18^O chronology through the use of ancient teak wood from archaeological sites.

## Methods

### Climate condition in northwestern Thailand

**Figure 5:**
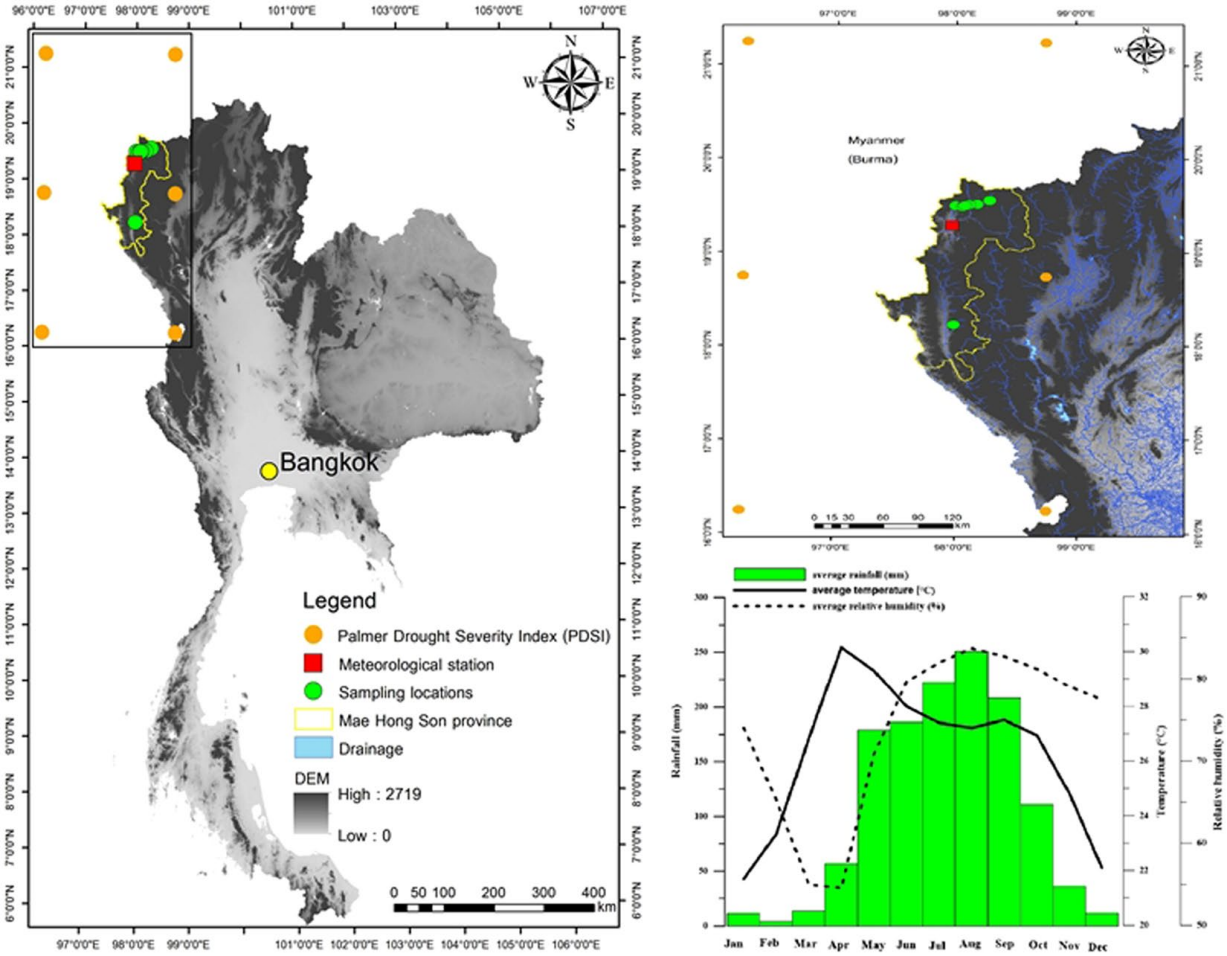
Study sites; orange circles represent six grids of PDSI, red square is the meteorological station Mae Hong Son, green circles are sampling locations, yellow line is the province boundary of Mae Hong Son, blue lines is drainage, gray scale is elevation (the left hand image), the expanded image PDSI (Top right corner) and mean (period from 1951–2015) climate conditions in Mae Hong Son Station, northwestern Thailand (Bottom right corner). The map of Thailand is obtained from the Information Center of the Faculty of Environment and Resource Studies, Mahidol University, Thailand. The six closest grid points of the PDSI, during the years 1948–2014 derived from https://rda.ucar.edu/dataset/ds299.0/. Maps are created using ARGIS10.5 software owner by the Faculty of Environment and Resource Studies, Mahidol University, Thailand.

Our sampling sites are located in the Mae Hong Son province, northwestern Thailand. According to instrumental records of the nearest climate station Mae Hong Son, which is located ca. 60 km away from the study sites in northwestern Thailand, the summer monsoon climate is dominant for approximately 160 days per year during the months of May to October. During the rainy season, monthly long-term (1951–2015) average rainfall is in the range of 128–249 mm and has a mean of 193 mm, with monthly mean temperatures ranging from 20–30 °C, with a seasonal mean of 27.7 °C. Relative humidity in the rainy season is within the range of 60–90%, with a mean of 80%. During the dry season, which lasts from November to April, the number of rainy days is as low as ca. 10 days, but the only days of rain within the latter part of this season occur in March and April. Monthly mean rainfall is about 10 mm on average. The monthly mean temperature in the dry season ranges from 30–35 °C, with a mean of 30 °C. Relative humidity in the dry season is between 40–60%, with a mean of only 35% (Fig. 5). The map of Thailand is obtained from the Information Center of the Faculty of Environment and Resource Studies, Mahidol University, Thailand. Detailed in Fig. 5 is created using ARGIS10.5 software owner by the Faculty of Environment and Resource Studies, Mahidol University, Thailand.

### Tree-ring material

In this study, the teak samples were collected in Mae Hong Son province in northwest Thailand. The seven individual study sites are located within a distance of 10–20 km and are all distributed in the natural forest. Generally, teak can grow well on well-drained soils within an elevation range of 200–800 m a.s.l^76^. The samples used in this study grew at altitudes of 300–600 m a.s.l. Ring width series were measured and cross-dated in an previous study^77^, indicating that the last tree ring was formed in 2009. To update the stable isotope chronology, we collected additional samples from six trees in 2015. The criteria for selecting the teak samples for stable isotope analysis from the previously measured ring-width data were as follows: 1) very old tree age, 2) cross dating was successful and age determination was correct, and 3) all annual ring boundaries were clearly visible. To ensure that the re-collected samples matched the previously analyzed ring-width series, we measured and cross-dated the ring-width series from the re-collected samples, through the use of standard dendrochrological techniques. For all samples, cross-correlations were checked using the COFECHA software^78^. Finally, 21 living teak trees, which collectively spanned the 338-year period from AD 1678–2015, were selected for the study. Instead of using the widely applied pooling method, where individual tree-rings of identical age belonging to several individuals are mixed to save time and resources^79,80^, we analyzed δ^18^O from the individual trees separately. This was done to ensure that the isotope series from trees of the different study sites were significantly correlated and to also allow a full control of the statistical characteristics of the final mean isotope chronology over its complete length.

### Cellulose extraction

Annual rings were cut from each core with a scalpel under a binocular microscope. Each sample was ground in a ball mill prior to extracting the α-cellulose^81^. Alpha-cellulose was extracted following the method described by Wieloch *et al*.^82^. Resin, fatty acids, etheric oils, and hemicellulose were extracted with a solution of 5% NaOH for 2 hours at 60 °C, two times. Then, lignin was extracted with 7% NaClO_2_ solution, plus 100% acetic acid for 8 hours at 60 °C, five times. For the final application, the solution should have a pH value of 4.5. The remaining hemicellulose was extracted with 17% NaOH for 2 hours at room temperature. A washing procedure with boiled deionized water was interposed for three times across the different steps. Finally, samples were treated once with 1% HCl for 5 min. at room temperature, rinsed with boiling deionized water three times and then transferred from the filter funnels into Eppendorf tubes with 1 ml deionized water.

The samples were then homogenized using an ultrasonic homogenizer and freeze dried for 72 hours in a lyophilisation unit. Approximately 300 µg of each dried α-cellulose sample were weighed and wrapped in silver foil to be processed in mass spectrometry and the δ^18^O was measured with an Elemental Analyzer coupled to a Delta V Advantage IRMS (Thermo Fisher), while laboratory standards were periodically interposed to test analytical replication. The δ^18^O values were referred from the International Standard (Vienna Standard Marine Ocean Water [VSMOW]), and the overall analytical precisions was ±0.3‰. Cellulose extraction was conducted at the Institute of Geography, University of Erlangen-Nuremberg, Erlangen, Germany.

### Statistical analyses

For evaluating coherence of these data, we calculated the mean inter-series correlation (Rbar)^83^ and the expressed population signal, or EPS^26^, which indicates how well the tree-ring δ^18^O (δ^18^O_tr_) estimates a theoretically infinite population. To determine the impact of the different climatic factors on δ^18^O_tr_, we computed simple correlations between δ^18^O_tr_ and monthly means of climate data. Climate data (temperature, rainfall, and humidity) were used from the Mae Hong Son climate station (1951–2015), which was about 60 km from the study region (hereinafter will be referred to as the local climate). Additionally, CRU TS4.03 (http://climexp.knmi.nl) gridded temperature/precipitation data, cover Mae Hong Son province 19°17′17″N, 97°57′52″E with a resolution 0.5° × 0.5° were used for correlation analyses. Henceforth, we will make use of the terminology “regional climate” for CRU-climate data. We also compared the δ^18^O rainfall of Bangkok (BKK, 1968–2015) and tested the similarity with various climatic indices: the Indian Monsoon (IM) Index = U850(40E-80E, 5N-15N)-U850(70E-90E, 20N-30N), the Western North Pacific Monsoon (WNPM) Index = U850(100E-130E, 5N-15N)-U850(110E-140E, 20N-30N), and the Webster and Yang Monsoon Index (WYM) Index = U850(40-110E,EQ-20N)-U200(40-110E, EQ-20N), all of which were accessed from http://iprc.soest.hawaii.edu/users/ykaji/monsoon/definition.html.

We analyzed the influence of different monsoon indices on our δ^18^O_tr_ series during the main ASM season (June–September) over the common period of 1948–2009. Furthermore, similarities with the Multivariate ENSO index (MEI) (https://www.esrl.noaa.gov/psd/enso/mei.ext/table.ext.html) and the Indian Ocean Dipole (IOD) model were investigated. The six closest grid points of the PDSI, during the years 1948–2014 (https://rda.ucar.edu/dataset/ds299.0/) were used for the evaluation of the climate sensitivity of δ^18^O_tr_. Furthermore, we employed the Royal Netherlands Meteorological Institute Climate Explorer (http://www.knmi.nl) to examine spatial correlations between δ^18^O_tr_ and sea surface temperatures (SSTs), which were obtained from the National Climatic Data Centre v3b data set.

The final precipitation reconstruction was derived by linear regression, and the validity was tested by calculating Pearson’s correlation coefficients and the variance explained, adjusted variance explained, Reduction of Error (RE), Coefficient Efficiency (CE), and Sign Test (ST)^30^. Precipitation over the past 338 years was then reconstructed using teak tree-ring δ^18^O. A Niño 4 index was used to test the stability of the teleconnection between the reconstructed precipitation and tropical pacific SSTs during the period 1870–2015. To extract cyclical variations in our records, we performed a spectral analysis on our δ^18^O_tr_ series using the REDFIT program for unevenly spaced time-series^84^ (Fig. S1).

## Supplementary information

Supplementary information.
