# Supplementary material for: A 338-year tree-ring oxygen isotope record from Thai teak captures the variations in the Asian summer monsoon system
Source: Sci Rep. 2020 Jun 2;10:8966. doi: 10.1038/s41598-020-66001-0 (PMC7265473; doi:10.1038/s41598-020-66001-0)
Supplement: Supplementary file 1 — Supplementary information. [file 41598_2020_66001_MOESM1_ESM.pdf]

Supplementary information for

A 338-year tree-ring oxygen isotope record from Thai teak captures the variations of the Asian summer monsoon system

Nathsuda Pumijumnong[1], Achim Bräuning[2], Masaki Sano[3], Takeshi Nakatsuka[4], Chotika Muangsong[5]*, Supaporn Buajan[1]

1) Faculty of Environment and Resource Studies, Mahidol University, Thailand, 2) Institute of Geography, Friedrich-Alexander University Erlangen-Nürnberg, Germany, 3) Faculty of Human Sciences, Waseda University, Tokorozawa, Japan, 4) Nagoya University, Japan, 5) Innovation for Social and Environmental Management, Mahidol University, Amnat Charoen Campus, Amnat Charoen, Thailand

*Corresponding author: Chotika Muangsong, chokyaom@hotmail.com, chotika.mua@mahidol.ac.th, Innovation for Social and Environmental Management, Mahidol University, Amnatcharoen Campus, Amnatcharoen 37000, Thailand

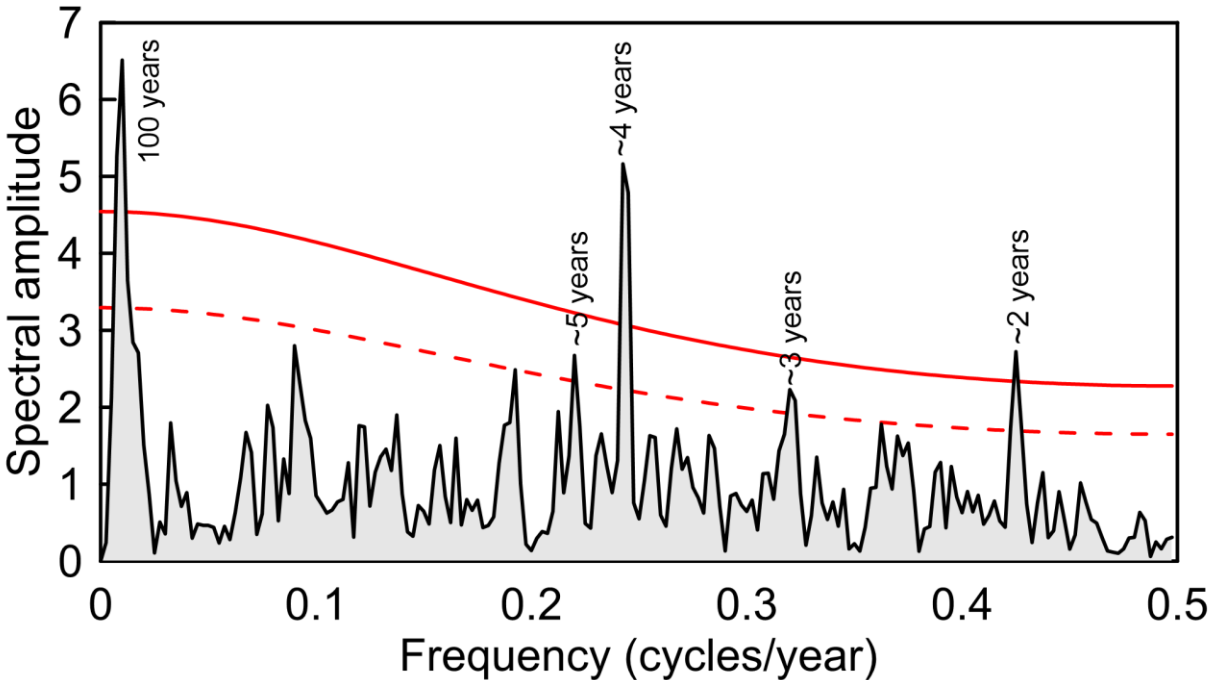

Figure S1 Spectral analysis of the $\delta^{18}O_{tr}$ series using the REDFIT. Grey shaded areas indicate the power spectrum of the $\delta^{18}O_{tr}$ series. The solid and dashed lines represent the 99% and 95% confidence limits, respectively, relative to the red-noise spectrum. The confidence limits were estimated using a Monte Carlo simulation. Significant peaks are shown for each period.

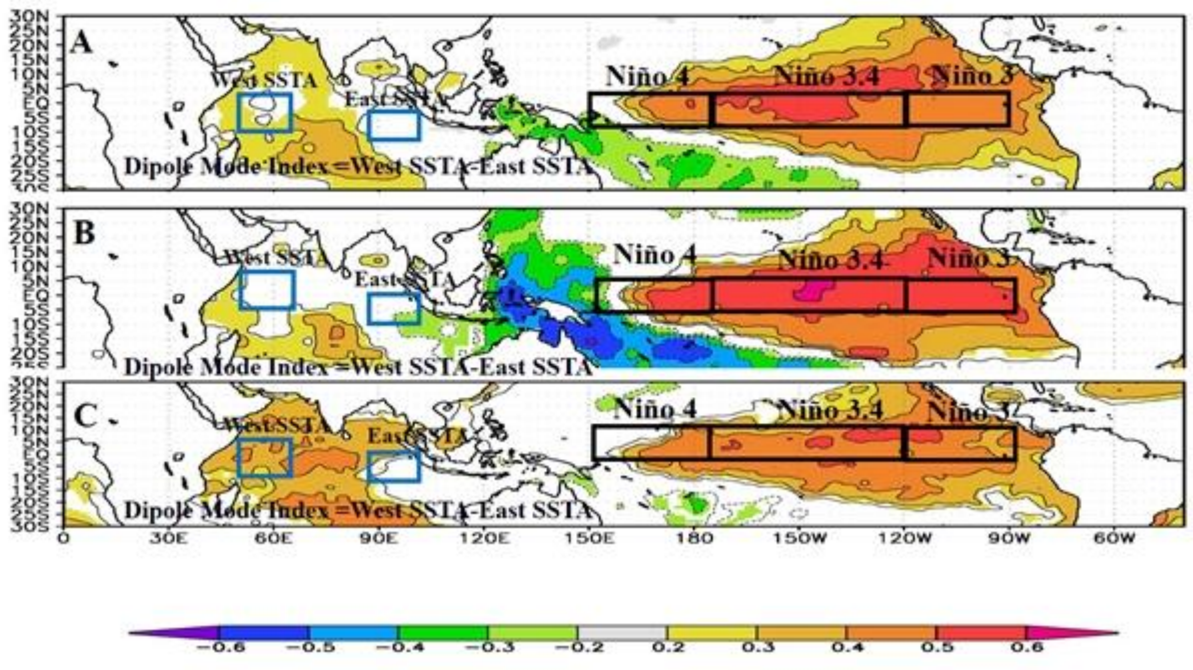

Figure S2 The correlation between average Teak Oxygen Isotope Values (May-December) and HadlSST (Niño3.4) in different time period (A) 1870-2015, (B) 1870-1942, (C) 1943-2015, the blue frames are West SSTA and East SSTA and the black frames are Niño4, Niño3.4, and Niño3.

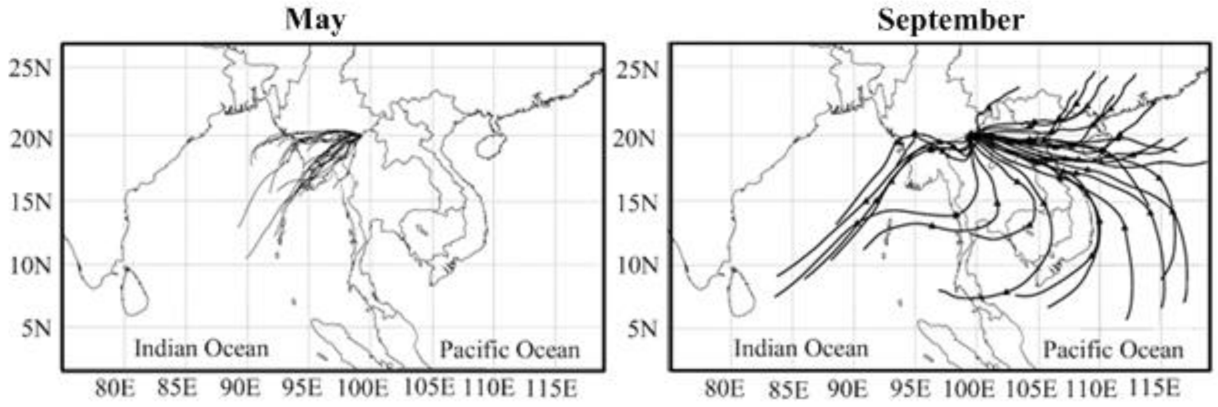

Figure S3 The 72-h back trajectories starting at 500, 1,000, and 1,500 m above ground level [1] for May and September at the study location.

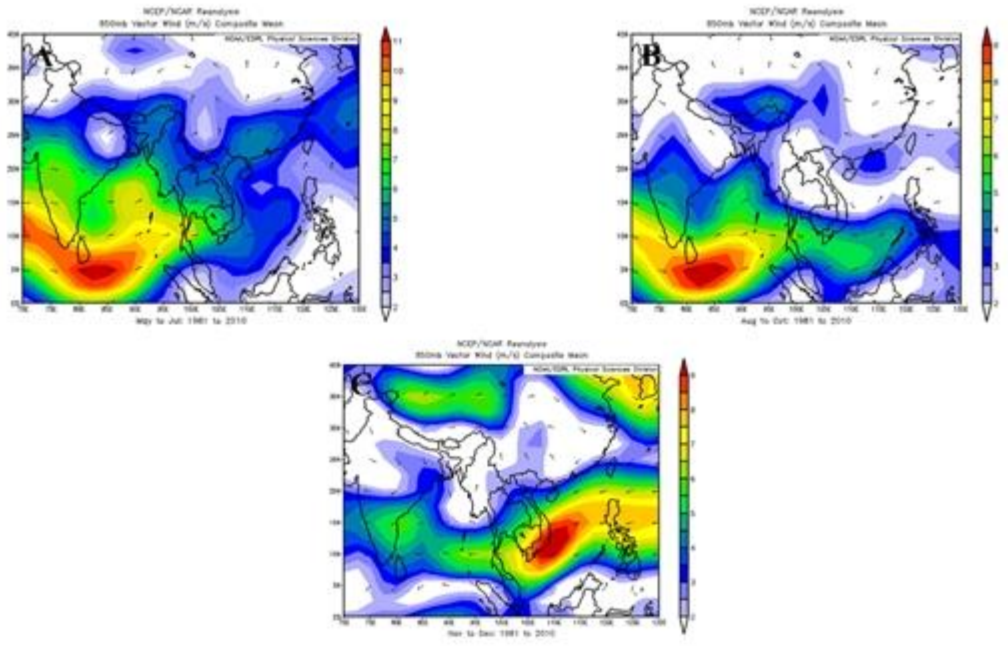

Figure S4 Composite averages of the 850-hPa wind vectors between AD 1981-2010 for May to July of (A), August to September (B), and November to December (C). The NCEP/NCAR wind reanalysis data were obtained from NOAA/ESRL Physical Sciences Division (PSD), Boulder, Colorado (http://www.esrl.noaa.gov/psd/).

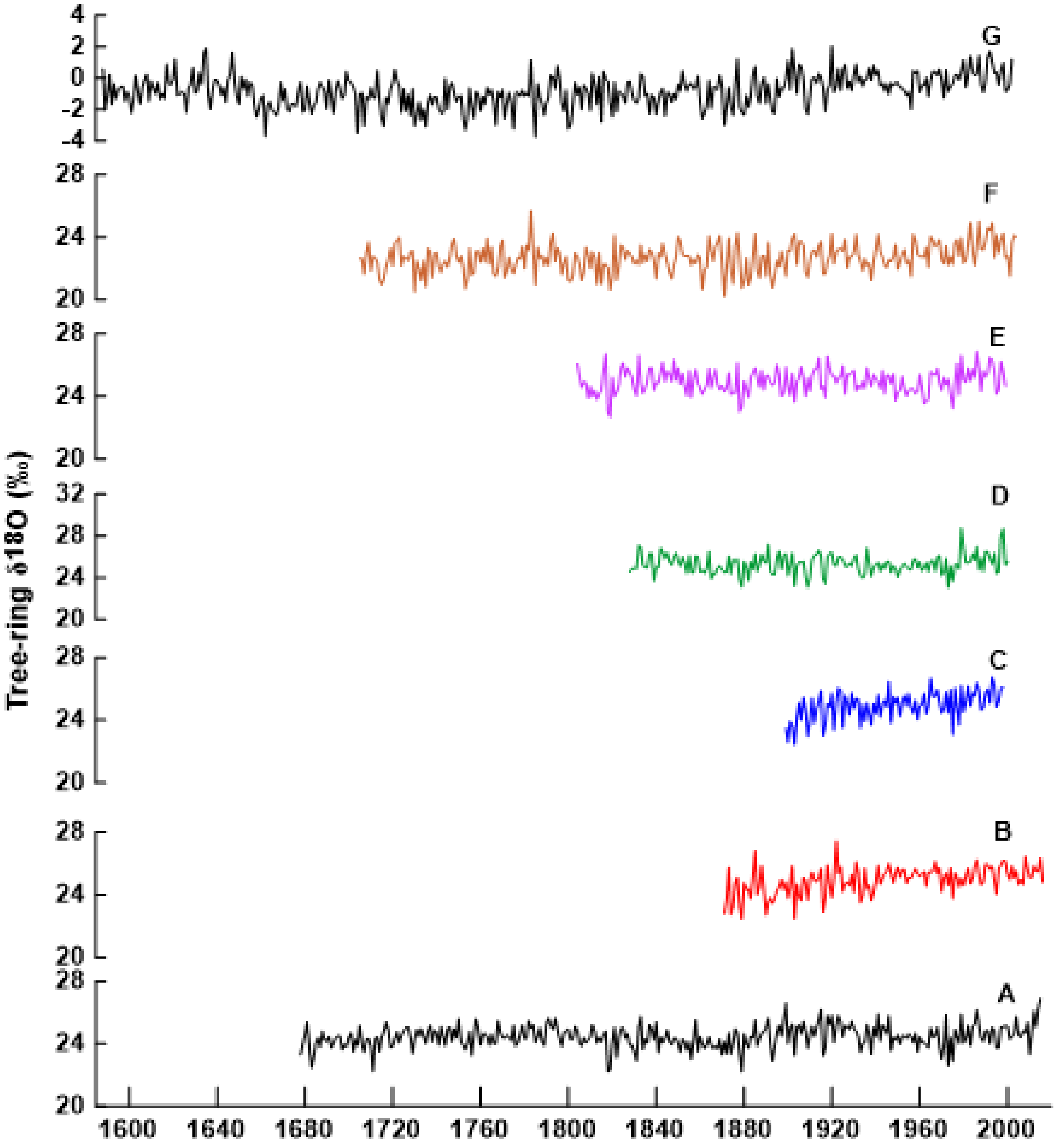

Figure S5 Comparisons of the oxygen isotope record of teak $\delta^{18}O_{tr}$ in this study (A) the oxygen isotope record of teak $\delta^{18}O_{tr}$ from Phrae province [2] (B), the oxygen isotope record of teak $\delta^{18}O_{tr}$ from Myanmar[3] (C), the oxygen isotope record of *Pinus merkusii* $\delta^{18}O_{tr}$ from Mae Hong Son [4] (D), the oxygen isotope record of *Pinus merkusii* $\delta^{18}O_{tr}$ from Uppang [5] (E), the oxygen isotope record of *Fokienia hodginsii* $\delta^{18}O_{tr}$ from Vietnam [6] (F), and the oxygen isotope record of *Fokienia hodginsii* $\delta^{18}O_{tr}$ from Laos [7] (G)

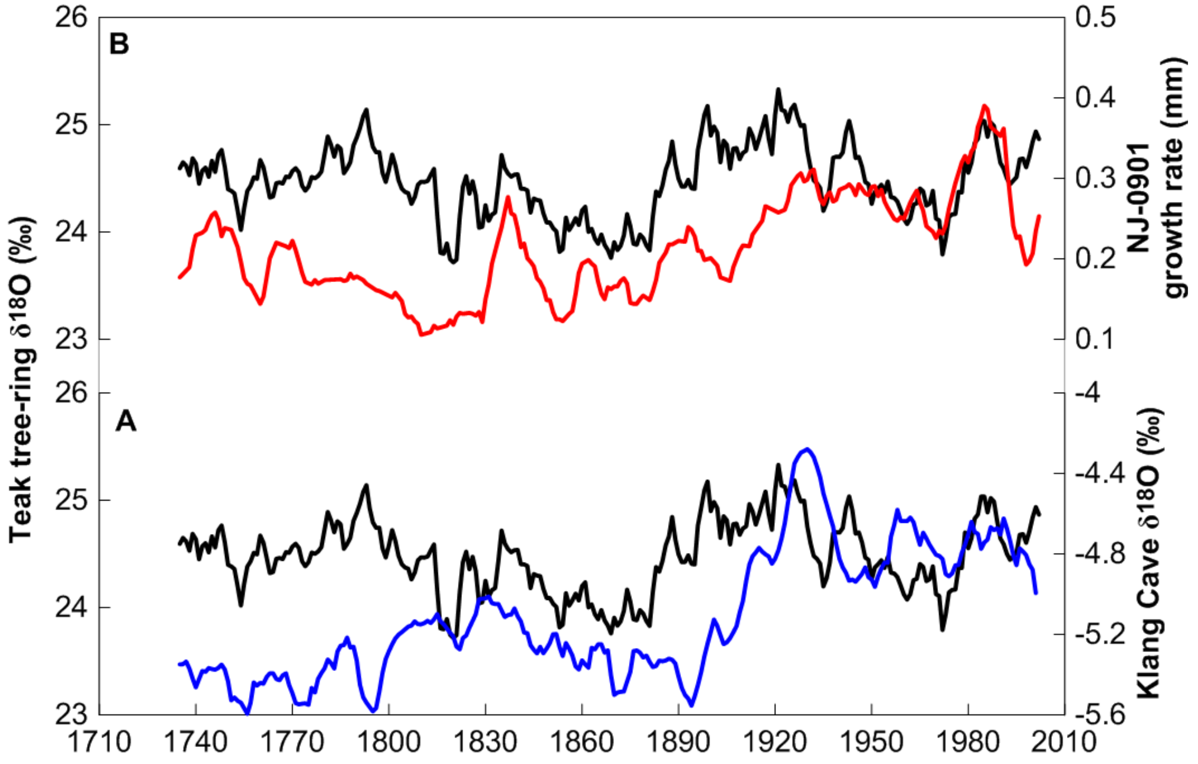

Figure S6 Comparisons of (A) the oxygen isotope record of stalagmites from Klang Cave in southern Thailand (blue line) [8] and (B) growth rate profile of stalagmite NJ-0901 from Namjang cave in Mae Hong Son province of northwestern Thailand (red line) [9] with the teak $\delta^{18}O_{tr}$ in this study (black line). All series were smoothed with a 7-point running-average filter.

References:

1. Stein, A., Draxler, R., Rolph, G. & Stunder, B. B., Cohen, MD, and Ngan, F.: NOAA'S HYSPLIT atmospheric transport and dispersion modeling system, B. *Am. Meteorol. Soc* **96**, 2059-2077 (2015).
2. Muangsong, C. *et al.* Effect of changes in precipitation amounts and moisture sources on inter-and intra-annual stable oxygen isotope ratios (δ18O) of teak trees from northern Thailand. *Agricultural and Forest Meteorology* **281**, 107820, doi:https://doi.org/10.1016/j.agrformet.2019.107820 (2020).
3. Pumijumnong, N., Muangsong, C., Buajan, S., Sano, M. & Nakatsuka, T. Climate variability over the past 100 years in Myanmar derived from tree-ring stable oxygen isotope variations in Teak. *Theoretical and Applied Climatology* **139**, 1401-1414, doi:https://doi.org/10.1007/s00704-019-03036-y (2020).
4. Xu, C., Pumijumnong, N., Nakatsuka, T., Sano, M. & Li, Z. A tree-ring cellulose δ18O-based July–October precipitation reconstruction since AD 1828, northwest Thailand. *Journal of Hydrology* **529**, 433-441 (2015).
5. Xu, C., Pumijumnong, N., Nakatsuka, T., Sano, M. & Guo, Z. Inter- annual and multi- decadal variability of monsoon season rainfall in central Thailand during the period 1804–1999, as inferred

from tree ring oxygen isotopes. *International Journal of Climatology* **38**, 5766-5776, doi:https://doi.org/10.1002/joc.5859 (2018).

6. Sano, M., Xu, C. & Nakatsuka, T. A 300- year Vietnam hydroclimate and ENSO variability record reconstructed from tree ringδ18O. *Journal of Geophysical Research: Atmospheres* **117** (2012).
7. Xu, C., Sano, M. & Nakatsuka, T. Tree ring cellulose δ18O of Fokienia hodginsii in northern Laos: a promising proxy to reconstruct ENSO? *Journal of Geophysical Research: Atmospheres* **116** (2011).
8. Tan, L. *et al.* Rainfall variations in central Indo-Pacific over the past 2,700 y. *Proceedings of the National Academy of Sciences* **116**, 17201-17206, doi:https://doi.org/10.1073/pnas.1903167116 (2019).
9. Muangsong, C., Cai, B., Pumijumnong, N., Hu, C. & Cheng, H. An annually laminated stalagmite record of the changes in Thailand monsoon rainfall over the past 387 years and its relationship to IOD and ENSO. *Quaternary International* **349**, 90-97, doi:https://doi.org/10.1016/j.quaint.2014.08.037 (2014).
